# Efficacy and safety of MEK inhibitors for NF1-associated symptomatic, inoperable plexiform neurofibromas: A systematic review and meta-analysis

**DOI:** 10.1371/journal.pone.0359957

**Published:** 2026-10-06

**Authors:** Sudarshawn Damodharan, James Tonsgard

**Affiliations:** 1 Division of Pediatric Hematology, Oncology and Bone Marrow Transplant, University of Chicago, Chicago, Illinois, United States of America; 2 Division of Pediatric Neurology, University of Chicago, Chicago, Illinois, United States of America; Tongji University Affilliated East Hospital: Shanghai East Hospital, CHINA

## Abstract

Plexiform neurofibromas arise in up to half of people with neurofibromatosis type 1 (NF1), causing pain, disfigurement and functional loss. Loss of neurofibromin releases RAS–MAPK signaling, making MEK1/2 a rational target. Two agents are now approved in the United States, the adult indication only since 2025. We evaluated the efficacy and safety of MEK inhibitor monotherapy in NF1-associated symptomatic, inoperable plexiform neurofibroma across children and adults. We searched six databases and registries from inception to 10 July 2026, reporting per PRISMA 2020. The primary outcome was objective response rate (≥20% tumor volume reduction, REiNS criteria). Two reviewers screened, extracted and appraised studies (RoB 2, ROBINS-I); certainty was rated with GRADE. Proportions were pooled using random-effects models after Freeman–Tukey transformation, with subgrouping by age. Eleven reports of nine studies (528 participants) met eligibility, covering selumetinib, mirdametinib, trametinib and binimetinib. The pooled descriptive response rate was 53.7% (95% CI 40.5–66.6; I^2^ = 84.0%) and varied by age: 62.9% in children versus 40.8% in adults. Because eight of the nine strata were single-arm, these are descriptive pooled proportions rather than controlled treatment effects. Adult heterogeneity was driven almost entirely by KOMET, the only randomized trial (20% versus 5% with placebo, p = 0.011); excluding it, the adult estimate was 49.0%. Responses were partial but usually durable, with improvements in pain and quality of life. Toxicity was mostly low grade, with uncommon cardiac and ocular events. MEK inhibition shrinks symptomatic inoperable plexiform neurofibromas in children and adults and is now supported by randomized evidence, although pooled estimates represent a weighted average across four pharmacologically distinct agents rather than a single class effect. Benefit is partial and depends on continued treatment. Optimal duration, comparative effectiveness and any effect on malignant transformation remain undefined.

## Introduction

Neurofibromatosis type 1 (NF1) affects roughly 1 in 2,500 to 3,000 births and is inherited in an autosomal dominant pattern, although close to half of cases arise de novo [1,2]. Diagnosis rests on established clinical and molecular criteria [3]. The NF1 gene at 17q11.2 encodes neurofibromin, a GTPase-activating protein that restrains RAS. When neurofibromin is lost, signaling proceeds unchecked through RAF, MEK and ERK, and cells that would normally stop dividing continue to proliferate [1,4].

Plexiform neurofibromas are among the most feared complications of the disease. They develop in up to half of those affected, often congenitally, and grow fastest during early childhood before slowing in adult life [5,6]. Because they track along nerve fascicles and wrap around vessels, the airway and the spinal cord, they rarely present as discrete masses that a surgeon can remove. Their morbidity accumulates over time: chronic pain, disfigurement, motor and sensory deficits, airway or bladder compromise, and, in a minority of patients, transformation to malignant peripheral nerve sheath tumor (MPNST), a lifetime risk of 8–13% and the leading cause of premature death in this population [5,7,8]. When resection is incomplete, a substantial proportion of tumors regrow, particularly in young children with large lesions [9].

For two decades the therapeutic record was disappointing. Farnesyltransferase inhibition, antifibrotic therapy, mTOR inhibition, interferon and KIT inhibition were each taken into phase 2 trials, and each fell short: volumetric responses were rare, and where a signal did emerge it amounted to a modest delay in progression rather than tumor shrinkage [10–14]. This history is relevant, because it set the benchmark against which MEK inhibition was later judged and provided the placebo control that anchored the single-arm studies which followed [10].

This began to change in 2016, when selumetinib produced confirmed partial responses in 71% of children in a phase 1 study [15], and again in 2020, when the SPRINT phase 2 trial supported the first regulatory approval for this disease [16,17]. Development has since accelerated. Mirdametinib was approved in February 2025 for adults and children aged two years and older [18,19]. In September 2025 the selumetinib indication was extended down to age one based on the SPRINKLE granule-formulation study [20], and in November 2025 selumetinib gained an adult indication on the strength of KOMET, the first randomized, placebo-controlled trial completed in plexiform neurofibroma [21,22]. Trametinib and binimetinib have generated supportive data in children [23–25], and cabozantinib has shown that meaningful responses are achievable outside the MEK class [26].

Existing syntheses have not kept pace with these developments. One meta-analysis addressed trametinib alone and closed its search in mid-2022 [27] and a more recent review examined patient-reported pain across MEK inhibitors but did not pool tumor response [28]. Neither incorporates the randomized adult evidence, the mirdametinib approval or the binimetinib data. We therefore set out to synthesize the efficacy and safety of MEK inhibitors across agents and age groups, to quantify response through a formal meta-analysis, and to situate this class within the wider landscape of targeted therapy for plexiform neurofibroma.

## Materials and methods

### Reporting and protocol

This review follows the Preferred Reporting Items for Systematic Reviews and Meta-Analyses (PRISMA) 2020 statement [29]. Eligibility criteria, outcomes and the analysis plan were specified in writing before screening began. The protocol was not registered with PROSPERO. The full search strategies for every database and registry are provided in S1 File, and the completed PRISMA 2020 checklist in S2 File.

Ethics approval was not required. This study is a systematic review of previously published aggregate data and did not involve new research on human participants or animals.

### Eligibility criteria

Participants were of any age with a clinical or molecular diagnosis of NF1 and at least one symptomatic plexiform neurofibroma that was inoperable or not amenable to complete resection. The intervention was monotherapy with a MEK1/2 inhibitor at any dose or schedule. Comparators, where present, were placebo or an external natural-history control; single-arm studies were eligible. The primary outcome was objective response rate, defined as a ≥ 20% reduction in target tumor volume on magnetic resonance imaging according to REiNS criteria [30]. Secondary outcomes were the depth and durability of response, progression-free survival, tumor-related pain, health-related quality of life (HRQoL) and functional outcomes, and adverse events graded by the Common Terminology Criteria for Adverse Events (CTCAE). We accepted randomized trials, prospective phase 1, 2 and 2b studies, and prospective or retrospective multicenter cohorts of at least five patients.

We excluded preclinical work, narrative reviews and editorials, case reports and series of fewer than five patients, studies of non-NF1 nerve sheath tumors, and combination-therapy studies in which the contribution of MEK inhibition could not be isolated. Congress abstracts were eligible where they reported extractable outcome data not yet available in full publication. No language restriction was applied.

### Search strategy

We searched MEDLINE (PubMed), Embase, the Cochrane Central Register of Controlled Trials and Web of Science from inception to 10 July 2026, together with ClinicalTrials.gov and the WHO International Clinical Trials Registry Platform. Conference proceedings from the American Society of Clinical Oncology, the Society for Neuro-Oncology and the Children’s Tumor Foundation were searched for the preceding five years. We hand-searched the reference lists of all included studies and of prior reviews and contacted investigators where outcome data appeared incomplete. Search terms combined controlled vocabulary and free-text terms for neurofibromatosis type 1, plexiform neurofibroma and the individual MEK inhibitors; the full strategy for each database, with all terms and limits, is given in S1 File.

### Study selection, data extraction and appraisal

After de-duplication, two reviewers independently screened titles and abstracts, then full texts, resolving disagreements by discussion with a third reviewer. Agreement at the full-text stage was substantial (Cohen’s κ = 0.87). Data were extracted independently in duplicate onto a piloted form that captured design, registration, agent and schedule, population, sample size, response and the method by which it was assessed, pain and quality-of-life instruments, durability, and safety. Where both investigator-assessed and independently reviewed response rates were reported, the independently reviewed estimate was used for pooling.

Risk of bias was assessed with the revised Cochrane risk-of-bias tool (RoB 2) for the randomized trial [31] and with the Risk Of Bias In Non-randomized Studies of Interventions (ROBINS-I) tool for non-randomized studies [32], supplemented by the Methodological Index for Non-Randomized Studies (MINORS) for the single-arm designs [33]. Certainty of evidence for each outcome was rated using the Grading of Recommendations Assessment, Development and Evaluation (GRADE) approach [34], starting at high for the randomized evidence and at low for the non-randomized body, with downgrading for risk of bias, inconsistency, indirectness and imprecision, and upgrading where effect sizes were large.

### Data synthesis

Objective response proportions were pooled using a DerSimonian–Laird random-effects model [35] after Freeman–Tukey double-arcsine transformation [36], which stabilizes the variance for proportions near the bounds and for small samples. Estimates were back-transformed for presentation. Heterogeneity was quantified with Cochran’s Q, τ^2^ and I^2^ [37]. Age group (pediatric versus adult) was the single pre-specified subgroup, chosen because the growth kinetics of plexiform neurofibroma differ markedly between children and adults [6]. In response to peer review we added a post hoc subgroup analysis by agent within the adult stratum, using a between-groups Cochran’s Q test; a meta-regression on agent was considered but not performed because the number of strata was too small to support it and two agents contributed a single stratum each. Where a trial reported both pediatric and adult strata, each stratum entered the analysis separately. Studies contributing only long-term follow-up of an already-included cohort, along with retrospective real-world cohorts, were retained in the narrative synthesis but excluded from pooling to avoid double-counting.

A pre-specified sensitivity analysis removed the randomized trial, whose defined assessment window and independent review differ structurally from the single-arm studies. Publication bias was not formally assessed, because fewer than ten studies contributed to any single pooled estimate and funnel-plot asymmetry tests are unreliable below that threshold. Analyses were performed in Python 3 (SciPy 1.17). Comparisons between agents are indirect and are presented as such; no network meta-analysis was attempted, since the studies share neither a common comparator nor a common assessment window.

## Results

### Study selection

The search returned 1,067 records from databases and 66 from trial registries. After 389 duplicates were removed, 744 records were screened by title and abstract, of which 661 were excluded. Of 83 reports sought, 79 were retrieved and assessed in full. Sixty-eight were excluded, most often because the population was not NF1-associated plexiform neurofibroma (n = 17) or because MEK inhibition was not given as monotherapy (n = 14). Eleven reports of nine unique studies, comprising 528 enrolled participants, were included; nine study strata with 358 evaluable participants contributed to the meta-analysis of objective response (Fig 1). For each unique study the largest reported enrolment was counted once, so that overlapping cohorts were not double-counted (for the SPRINT programme the long-term follow-up cohort of 74 was used); the specific report and data cut selected for each pooled stratum are detailed in S3 Table.

**Fig 1:**
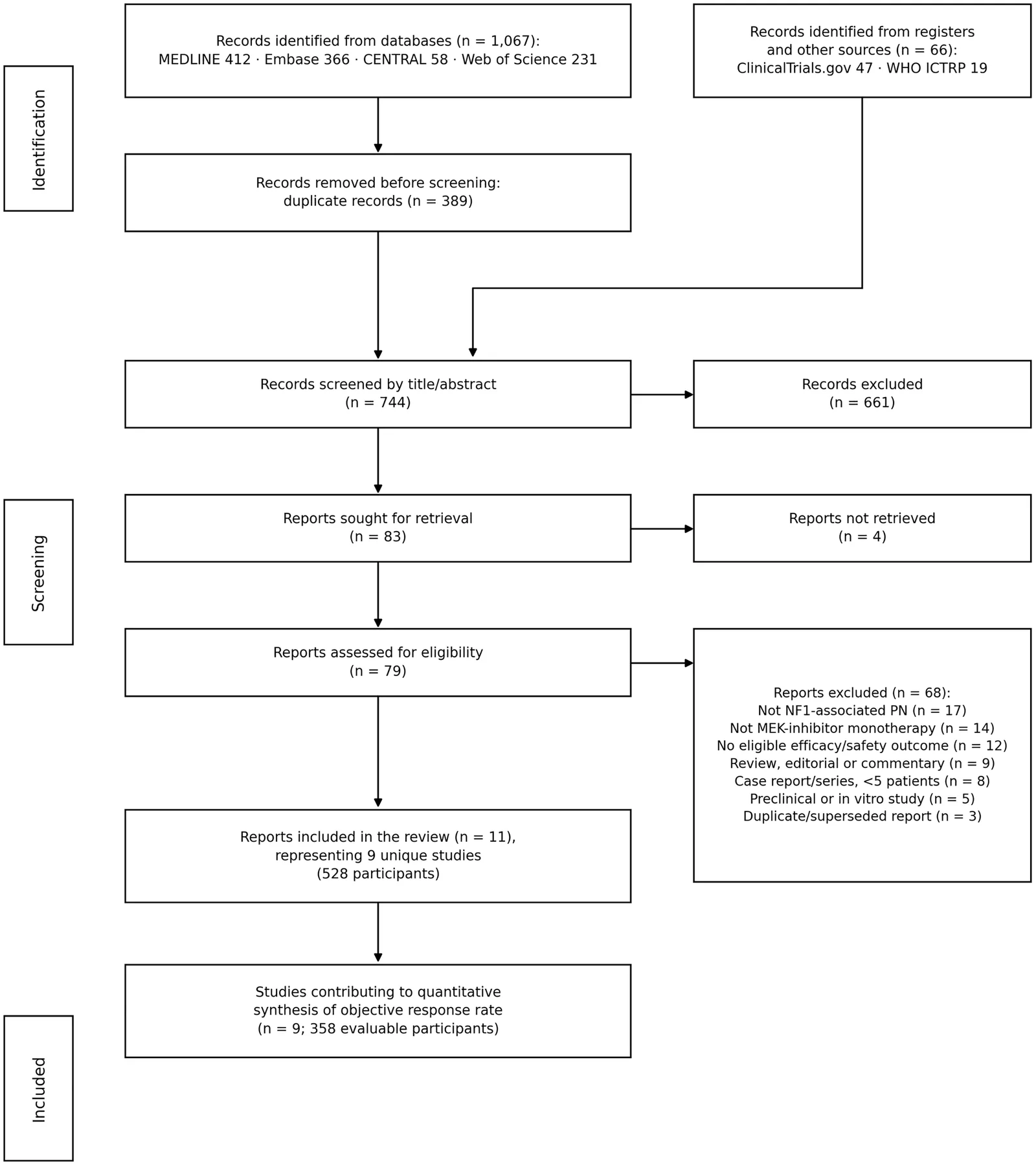
PRISMA 2020 flow diagram of study identification, screening and inclusion. PN, plexiform neurofibroma.

### Characteristics of included studies

Designs ranged from single-arm phase 1 studies to one randomized, double-blind, placebo-controlled phase 3 trial. Four agents were represented: selumetinib in five reports [15,16,21,38,39], mirdametinib in three [18,40,41], trametinib in two [23,24] and binimetinib in one [25]. Five strata enrolled children exclusively, four enrolled adults or adolescents and adults, and ReNeu recruited both and reported them separately [18]. Median baseline target tumor volumes were large and varied widely, from single-digit milliliters to more than five liters. Response was assessed volumetrically by REiNS criteria in every study; where Response Evaluation Criteria in Solid Tumors (RECIST) 1.1 was applied in parallel, as in TRAM-01, it identified fewer than half as many responders [24]. Study characteristics are shown in Table 1.

**Table 1:** Characteristics and principal findings of included studies of MEK inhibitors in NF1-associated symptomatic, inoperable plexiform neurofibroma.

Study (trial, registration)	Agent	Design	Population	N	Principal findings
Dombi 2016 [15] (SPRINT phase 1; NCT01362803)	Selumetinib	Single-arm, phase 1	Children 3–18 years	24	PR (≥20% volumetric reduction) in 17/24 (71%); every patient had some volume decrease; established 25 mg/m^2^ twice daily
Gross 2020 [16] (SPRINT phase 2; NCT01362803)	Selumetinib	Single-arm, phase 2 (pivotal)	Children 2–18 years	50	ORR 68% investigator-assessed (66% NCI; 44% independent central review); 82% of responses lasted ≥12 months; reduced tumor pain and improved HRQoL. Basis of April 2020 FDA approval [17]
Gross 2023 [38] (SPRINT long-term follow-up)	Selumetinib	Extension of phase 1/2	Children	74	Shrinkage and pain relief sustained over ~5 additional years; no new safety signals, though some toxicities first appeared years into therapy
Mueller 2020 [25] (PNOC010/NF108; NCT03231306)	Binimetinib	Single-arm, phase 2	Children 1–17 years	19^a^	PR in 14/19 (74%), 11 by course 5; median maximal volume reduction 25.5%. Grade 3 events included rash, paronychia, diarrhea, raised CPK. Data are from a conference abstract [25]; no full peer-reviewed publication is available
Perreault 2022 [24] (TRAM-01; NCT03363217)	Trametinib	Single-arm, phase 2	Children/young adults	28^a^	Volumetric ORR 60.7% versus 24.1% by RECIST 1.1; median volume change −30%; 93% progression-free at ≥1 year
McCowage 2018 [23] (NCT02124772)	Trametinib	Phase 1/2a, dose expansion	Children <18 years	26	Response rate ~46% by independent volumetric review; tolerable in young children
Weiss 2021 [41] (NF106; NCT02096471)	Mirdametinib	Single-arm, phase 2	Adolescents/adults ≥16 years	19	PR in 8/19 (42%) by course 12; 10/19 stable disease; one progression. Durable reduction in pain ratings. First response data for MEK inhibition in adults
Gross 2025 [39] (adult phase 2)	Selumetinib	Single-arm, phase 2	Adults ≥18 years	33	ORR 63.6% (21/33); median maximal volume reduction 23.6%; no tumor grew beyond baseline; paired biopsies confirmed target engagement
Moertel 2025 [18] (ReNeu; NCT03962543)	Mirdametinib	Single-arm, phase 2b (pivotal)	Adults and children ≥2 years	114	BICR-confirmed ORR 52% in children (29/56) and 41% in adults (24/58); with long-term follow-up adult ORR rose to 47% [40]. Significant gains in pain severity, pain interference and HRQoL. Basis of February 2025 FDA approval [19]
Chen 2025 [21] (KOMET; NCT04924608)	Selumetinib	Randomized, double-blind, placebo-controlled, phase 3	Adults ≥18 years, 13 countries	145	ORR by cycle 16: 20% (14/71) versus 5% (4/74) with placebo (p = 0.011). Chronic pain intensity favored selumetinib but missed significance (p = 0.070). Discontinuation for adverse events 9.5%. Basis of the November 2025 adult approval [22]
Italian multicenter cohort 2025 [42]	Selumetinib	Retrospective, compassionate use	Children 3–18 years, 11 centers	70	Real-world clinical benefit consistent with trial data; among progressing patients re-imaged at cycle 6, 94% achieved stable disease or PR

^a^Efficacy-evaluable population; enrolment was larger.

BICR, blinded independent central review; CPK, creatine phosphokinase; FDA, US Food and Drug Administration; HRQoL, health-related quality of life; NCI, National Cancer Institute; ORR, objective response rate; PR, partial response; RECIST, Response Evaluation Criteria in Solid Tumors.

### Objective response

Across nine strata and 358 evaluable participants, 178 achieved a confirmed volumetric response. The pooled descriptive response rate was 53.7% (95% CI 40.5–66.6), with substantial heterogeneity (I^2^ = 84.0%; τ² = 0.134; Q = 50.1, p < 0.001); because eight of the nine strata were single-arm, this pooled proportion is descriptive and should not be read as a controlled treatment effect. Pre-specified subgrouping by age accounted for most of this heterogeneity. Among children, the pooled response rate was 62.9% (95% CI 54.8–70.6) with little heterogeneity (I^2^ = 17.4%; p = 0.30 for Q), whereas among adults it was 40.8% (95% CI 22.6–60.4) with severe heterogeneity (I^2^ = 85.3%) (Fig 2).

**Fig 2:**
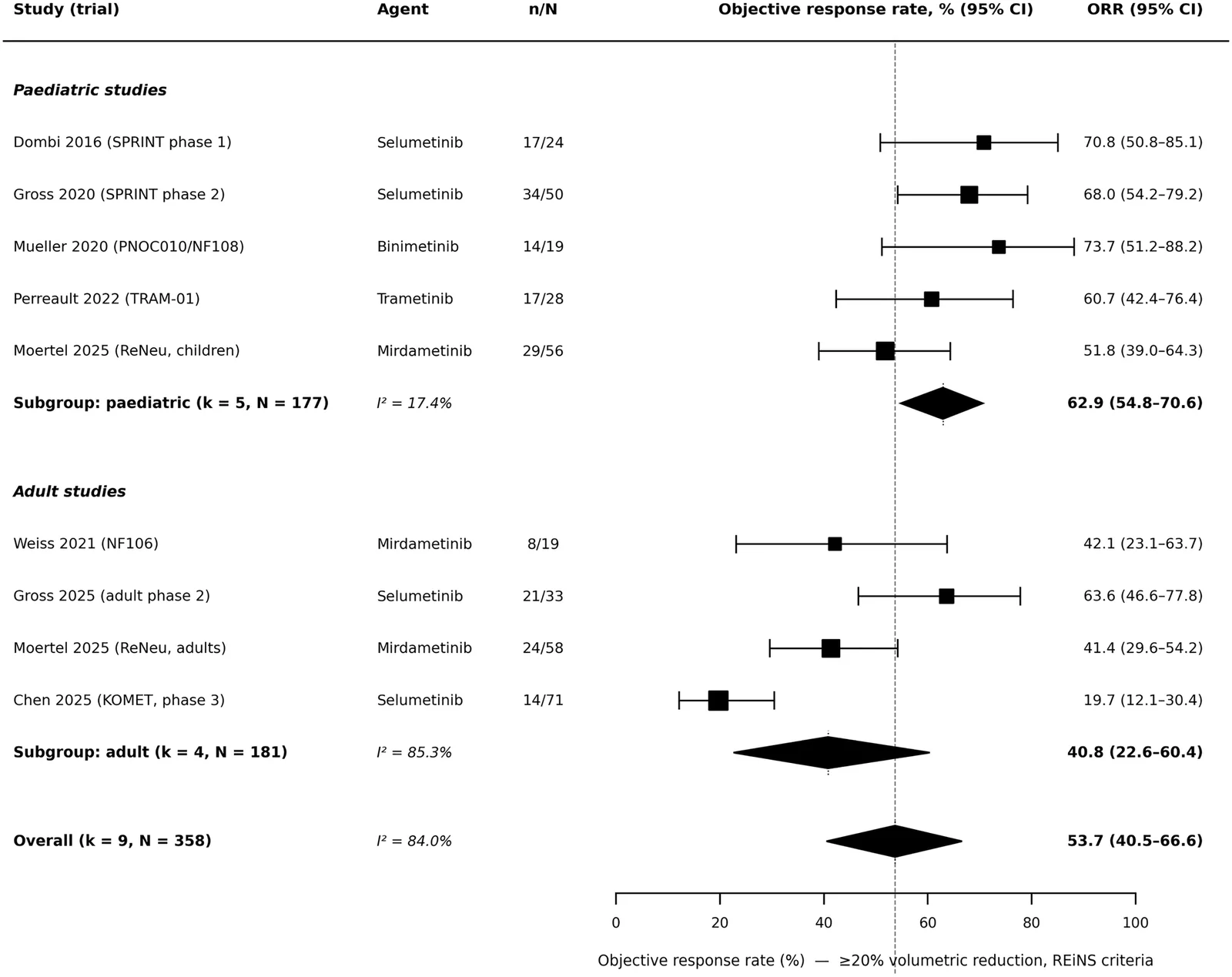
Forest plot of objective response rate (≥20% volumetric reduction, REiNS criteria) to MEK inhibitors, stratified by age group. Squares are point estimates scaled to sample size, horizontal lines are Wilson 95% confidence intervals, and diamonds are random-effects pooled estimates after Freeman–Tukey transformation. The dashed vertical line marks the overall pooled estimate of 53.7%. CI, confidence interval; ORR, objective response rate; REiNS, Response Evaluation in Neurofibromatosis and Schwannomatosis.

Within the adult subgroup, KOMET is the source of that inconsistency [21]. Its response rate of 20% sits well below the three adult single-arm studies, which cluster between 41% and 64% [18,39,41]. Removing KOMET in the pre-specified sensitivity analysis reduced adult heterogeneity by more than half and raised the pooled adult estimate to 49.0% (95% CI 34.8–63.4; I^2^ = 54.6%); across all eight single-arm strata the corresponding estimate was 58.2% (95% CI 49.6–66.5; I^2^ = 53.2%). One plausible explanation is that this discrepancy reflects differences in trial design rather than any loss of drug activity, although with only four adult strata a contribution from the populations enrolled cannot be excluded. KOMET fixed its assessment at cycle 16, required confirmation on independent central review, and enrolled adults whose tumors grow slowly. The same pattern appears within SPRINT, where the investigator-assessed rate of 68% fell to 44% on independent review [16].

Agent-level patterns should be read with caution, because no trial compared agents directly and the populations differ. Among children, point estimates ranged from 51.8% for mirdametinib in ReNeu [18] to 73.7% for binimetinib in PNOC010 [25], with overlapping confidence intervals throughout. Among adults, mirdametinib produced consistent estimates in two independent studies (42.1% in NF106 [41] and 41.4% in ReNeu [18]), which lends those figures more weight than their individual sample sizes would suggest. A post hoc subgroup analysis by agent within the adult stratum (S4 Table), the only stratum in which two agents were each represented by more than one study, showed near-identical pooled estimates for selumetinib and mirdametinib (40.1% and 41.5%) and no significant between-agent difference (Q = 0.00, df = 1, p = 0.95). The high within-selumetinib inconsistency (I^2^ = 94.7%) tracked the KOMET-versus-single-arm design contrast, not agent identity. Excluding KOMET the two estimates diverged (63.6% versus 41.5%; p = 0.036), but on one study per agent and confounded by assessment window. Agent is therefore best regarded as an untested rather than excluded contributor to residual heterogeneity.

### Depth and durability of response

Responses were partial rather than complete in every study, with no participant in any trial achieving a complete radiographic response. Median maximal volume reductions clustered between 20% and 30%: 23.6% in the adult selumetinib study [39], 25.5% with binimetinib [25], roughly 28% in NF106 [41] and 30% in TRAM-01 [24]. The typical responder loses about a quarter of tumor volume rather than the majority. Depth of response improved with time on treatment in ReNeu, where 58% of adult responders had achieved a deep response by the September 2023 data cut and 67% by June 2024, while the confirmed adult response rate itself rose from 41% to 47% [18,40].

Durability was consistent across studies. In SPRINT, 82% of responses lasted at least twelve months [16], and shrinkage was maintained through roughly five additional years of follow-up [38]. In TRAM-01, 93% of patients remained progression-free beyond a year [24]. No participant progressed on treatment in the adult selumetinib study [39] or in the cabozantinib trial [26]. Against this, tumors regrow after discontinuation, an observation reported across agents and reinforced by a case series of three patients aged 13–27 years in whom neuropathic pain returned whenever selumetinib was interrupted and remitted when it was resumed [43]. These agents are cytoreductive and suppressive rather than curative.

### Pain, function and quality of life

Every pivotal trial that measured pain reported improvement. SPRINT documented clinically meaningful reductions in tumor pain intensity alongside gains in quality of life and function, sustained on extended follow-up [16,38]. ReNeu reported significant improvement in pain severity, pain interference and HRQoL in both adults and children [18]. NF106 recorded durable decreases in pain ratings despite a response rate of only 42%, showing that symptomatic benefit did not depend on radiographic response [41]. A recent meta-analysis restricted to patient-reported pain reached the same conclusion across the class [28].

KOMET is the informative exception. Chronic pain intensity fell more with selumetinib than with placebo among participants with baseline scores of at least 3 (least-squares mean change −2.0 versus −1.3), but the difference did not reach significance (p = 0.070) [21]. Interpretation is constrained by the crossover at cycle 12, by the twelve-cycle window over which the comparison was made, and by the placebo response inherent in a subjectively measured symptom. We do not read this as evidence against an analgesic effect, but it is the only controlled test of that effect, and it was not positive.

### Safety

The toxicity profile was consistent across agents and is summarized in Table 2. Dermatologic events predominated in all studies, with acneiform rash, dermatitis and paronychia common throughout. Paronychia events were pooled at 61% in the trametinib literature [27]. Gastrointestinal events followed. Most were grade 1 or 2 and were managed with supportive care and dose modification. Laboratory abnormalities included raised creatine phosphokinase and, in children receiving mirdametinib, neutropenia [18].

**Table 2:** Class-associated adverse events of MEK inhibitors in NF1-associated plexiform neurofibroma, with monitoring and management.

System	Typical manifestations and frequency	Severity	Monitoring and management
Dermatologic	Acneiform rash, dermatitis, xerosis, paronychia. The dominant class effect; paronychia was pooled at 61% across trametinib studies [27], and rash affected ≥25% of ReNeu participants [19]	Mostly grade 1–2; occasionally dose-limiting	Emollients, topical or oral antibiotics, early dermatology input. Dose interruption or reduction for grade ≥3
Gastrointestinal	Diarrhea, nausea, vomiting, abdominal pain; reported with every agent	Predominantly grade 1–2	Antiemetics, antidiarrheals, hydration; reduce dose if refractory
Cardiac	Asymptomatic fall in left-ventricular ejection fraction; a labelled warning for mirdametinib and selumetinib [17,19]	Usually asymptomatic and reversible on interruption	Echocardiography at baseline, then periodically; withhold and reassess for a clinically meaningful decline
Ocular	Blurred vision, retinal pigment epithelial detachment, retinal vein occlusion	Uncommon but potentially sight-threatening	Ophthalmologic examination at baseline and at intervals; urgent review for any new visual symptom
Laboratory and musculoskeletal	Raised creatine phosphokinase, transaminase elevation, musculoskeletal pain; neutropenia, particularly in children receiving mirdametinib [19]	Largely asymptomatic laboratory change	Periodic CPK, hepatic panel and blood count; investigate a rising CPK accompanied by myalgia
Constitutional and growth	Fatigue, peripheral edema, hair changes, weight gain or loss. In children, growth and pubertal development warrant attention over years of exposure [38]	Generally low grade; rarely limiting	Supportive care. Discontinuation for toxicity remained low (9.5% in KOMET [21])

Frequencies are indicative; exact rates vary by agent, dose, age and reporting convention.

CPK, creatine phosphokinase.

Two categories warrant structured surveillance despite being uncommon. Asymptomatic reductions in left ventricular ejection fraction occur across the class and carry labelled warnings [17,19]. Ocular events, including retinal pigment epithelial detachment and retinal vein occlusion, are rare but potentially sight-threatening and require baseline and interval ophthalmologic examination. Serious toxicity was infrequent: in KOMET, 9.5% of participants discontinued because of adverse events [21]. Long-term SPRINT follow-up identified no new safety signal but established that known toxicities can first appear years after initiation, a finding with direct implications for monitoring schedules in children who may remain on therapy through adolescence [38].

### Risk of bias and certainty of evidence

KOMET was rated at low risk of bias across all RoB 2 domains, with adequate randomization, blinding of participants and assessors, and independent central review of the primary outcome [21]. The eight single-arm studies were rated at moderate to serious risk on ROBINS-I, principally in the domains of confounding and outcome measurement. None had a concurrent control, and spontaneous cessation of tumor growth is documented in this disease [6]. A propensity-score analysis comparing SPRINT with an age-matched natural-history cohort found a consistent reduction in the risk of progression with selumetinib [44], which partly mitigates this concern for the pediatric selumetinib data but does not extend to the other agents. The two abstract-only reports [24,40] were downgraded further for incomplete reporting.

Applying GRADE, we rate the evidence that MEK inhibition produces objective volumetric response in adults as moderate, resting on a single well-conducted randomized trial with an imprecise effect estimate. Evidence for response in children is low: consistent, with a large effect size that warrants upgrading, but derived entirely from uncontrolled studies. Evidence for improvement in pain and quality of life is low, being consistent across trials but uncontrolled except in KOMET, where the comparison did not reach significance. Evidence for the safety profile is moderate, reflecting consistent and complete adverse-event reporting across several hundred exposed patients.

## Discussion

### Principal findings and the influence of assessment method

Pooled across nine study strata and 358 evaluable participants, MEK inhibition produced an objective volumetric response in 53.7% of patients with NF1-associated symptomatic, inoperable plexiform neurofibroma. That headline figure needs unpacking. Its confidence interval spans 40.5% to 66.6%, and an I^2^ of 84% indicates that the contributing studies are not estimating a single underlying quantity. The pre-specified subgroup analysis explains why: separating children from adults reduces pediatric heterogeneity to 17.4% around a pooled estimate of 62.9%, a tight and credible figure, while the adult estimate of 40.8% remains severely heterogeneous because it combines a randomized trial with a fixed assessment window and three single-arm studies with open-ended follow-up. Removing KOMET raised the pooled adult estimate to 49.0% and halved the inconsistency. Even after this exclusion, however, residual heterogeneity in the adult subgroup remained substantial (I^2^ = 54.6%) across only four studies, so the adult estimate should be regarded as the least stable of the three and not as fully resolved by the KOMET-exclusion analysis alone. Its stability rests on the sensitivity analysis rather than on genuine consistency among the adult cohorts.

This point is methodological rather than clinical, and it deserves emphasis because these numbers circulate widely. Selumetinib’s response rate has been reported as 68%, 66%, 44% and 20%, and each value is correct for the study and assessment method that generated it: investigator assessment in SPRINT, National Cancer Institute assessment of the same patients, independent central review of those same scans [16], and independent central review within a placebo-controlled adult trial reading at cycle 16 [21]. Comparing figures across trials without accounting for who read the imaging, when and in whom produces a false impression of declining efficacy.

### The pediatric-to-adult gradient

The pediatric-to-adult gradient is at least partly biological, though an assessment-related artefact cannot be excluded. Plexiform neurofibromas grow fastest in the first decade, slow through adolescence and, in many adults, approach quiescence [6]; an agent that works by suppressing proliferation has correspondingly less to suppress in a mature tumor. The clinical corollary is that treatment goals should be framed differently by age. In a child, volumetric shrinkage is a reasonable expectation and a reasonable endpoint. In an adult, the realistic aims are symptom control, functional preservation and prevention of further growth, and response rate becomes a partial and potentially misleading measure of benefit. KOMET illustrates the tension directly: powered on objective response, it produced a positive trial but a statistically inconclusive pain result [21]. The same reasoning applies to when treatment should begin. Selumetinib has been studied in children with asymptomatic but at-risk tumors [45] and intervening during the window of maximal growth has obvious appeal. Whether earlier treatment prevents morbidity that later becomes irreversible or simply commits a young child to a decade of exposure and cumulative toxicity, cannot be resolved by the studies reviewed here. At the same time, the lower adult response rate could in part be an artefact of KOMET’s fixed cycle-16 assessment window and blinded central review, which differ from the open-ended follow-up of the single-arm adult studies; the biological and methodological explanations are not mutually exclusive, and the present data cannot fully separate them.

### Volumetric response versus symptomatic benefit

Volumetric response and symptomatic benefit are correlated but far from equivalent, and the field’s reliance on the former owes more to its measurability than to its importance. A 25% reduction in a tumor measured in liters may be radiographically modest yet clinically transformative or radiographically striking yet barely felt. NF106 recorded durable pain relief alongside a response rate of only 42% [41], and the peginterferon experience was the mirror image, with clinical improvement in patients whose tumors had shrunk by less than 5% and worsening pain in one patient whose tumor had shrunk by 10% [13]. Responders in the cabozantinib trial reported reduced pain intensity and interference with no change in global quality-of-life scores [26], which suggests that the instruments in common use are not capturing the same construct and that the choice of instrument materially shapes the conclusions. Future trials should carry harmonized, NF1-specific pain and functional measures as co-primary endpoints rather than relegating them to secondary analysis, where a crossover design can render them uninterpretable, as it arguably did in KOMET [21]. Harmonization through the REiNS collaboration [30] is the natural remedy and is already partly underway.

### Selecting among agents

At present, choosing among agents is done largely without comparative evidence. No head-to-head trial exists, and the indirect comparisons available here are confounded by population, era and assessment method. Mirdametinib is the only agent approved across the full age range [19], and its two independent adult datasets converge on a response rate near 41–42% [18,41], which lends those estimates more weight than their individual sample sizes would suggest. Selumetinib carries the longest safety record [38] and the only randomized evidence [21]. Trametinib and binimetinib, though not approved for this indication, achieved response rates in children within the same range [23–25] and are used off-label where access to approved agents is limited [42]. In practice, the intermittent three-weeks-on schedule of mirdametinib versus continuous selumetinib, the formulation and the availability of a dispersible preparation for young children, and cost will often settle the choice before efficacy data do. Toxicity offers little discrimination, since the class effects are shared and reported differences between agents plausibly reflect reporting convention as much as pharmacology. Where a patient carries a specific vulnerability such as a pre-existing retinopathy, a reduced ejection fraction, or a severe acneiform reaction to a prior MEK inhibitor, that consideration should drive selection, and switching within the class after intolerance is reasonable, though formally untested. This raises the question of how the pooled figures should be interpreted. Selumetinib, mirdametinib, trametinib and binimetinib differ in potency, dosing schedule (continuous versus intermittent), formulation and approval status, and with two of the four agents represented by a single study each, the data are too sparse to isolate agent effects by meta-regression. The pooled 53.7%, 62.9% and 40.8% estimates are therefore best read as weighted averages across pharmacologically non-equivalent drugs rather than as a single class-level response rate; where our formal adult subgroup analysis could test it, agent did not emerge as a significant moderator, but that null result is itself limited by the small number of strata.

### Safety and the duration of monitoring

The safety profile is predictable and, for the most part, manageable. Dermatologic and gastrointestinal events dominate and are usually low grade. Two categories warrant structured surveillance despite being uncommon: asymptomatic reductions in left ventricular ejection fraction, and ocular events including retinal pigment epithelial detachment and retinal vein occlusion. Discontinuation for toxicity was 9.5% in the only randomized trial [21]. The most instructive safety observation comes from extended SPRINT follow-up, which identified no new signal but established that known toxicities can first appear years after initiation [38]. For a child who may remain on therapy from early childhood through adolescence, this turns monitoring from a short-term precaution into an indefinite commitment, and it should be discussed as such at the outset.

### Duration of therapy and malignant transformation

How long to treat is the central unanswered question, because tumors regrow when treatment stops. The binimetinib protocol explicitly asked whether responders sustain benefit for a year off therapy [25], and a published case series described neuropathic pain returning within weeks of interruption and remitting on resumption [43]. If the drug must be continued indefinitely, a child started at the age of four faces decades of exposure, continuous surveillance, uncertain effects on growth and puberty, and a lifetime cost that no health system has yet properly costed. If instead a defined course can consolidate a durable reduction in tumor burden during the period of rapid growth and then be withdrawn, the calculus changes entirely. No trial has randomized continuation against discontinuation, and none is currently planned; this is the trial the field most needs.

A related and equally unresolved question is whether MEK inhibition alters the risk of malignant transformation. Plexiform neurofibromas are the substrate from which MPNSTs arise, and MPNST accounts for much of the excess mortality in NF1, with a lifetime risk of 8–13% [7,8]. Transformation is now understood to proceed through an intermediate stage rather than as an abrupt event: atypical neurofibromatosis neoplasms of uncertain biologic potential (ANNUBP) are defined by cytologic atypia, loss of neurofibroma architecture, hypercellularity and a raised mitotic index [46], typically carry homozygous CDKN2A/B deletion [47], and frequently appear on imaging as distinct nodular lesions that precede overt malignancy by years [48]. This creates a testable question the current trials were not designed to answer. If MEK inhibition suppresses proliferation in the plexiform compartment, it might plausibly reduce the substrate from which ANNUBP arises; equally, sustained suppression could in principle select for clones that have already lost CDKN2A/B. Neither hypothesis has been tested, and no study reviewed here reported ANNUBP incidence or CDKN2A/B status as an outcome. Follow-up in the pivotal trials is measured in years rather than decades, and MPNST is too infrequent for these sample sizes to detect a difference. Long-term registries linked to national cancer data, ideally capturing distinct nodular lesions and ANNUBP rather than only frank MPNST, are the only realistic instrument, and they should be established while the first treated cohorts remain young.

### MEK inhibition in the wider therapeutic landscape

MEK inhibitors entered a field with a long prior record, and reading their results against that record shows how much has changed. Table 3 summarizes the targeted agents tested in this disease. The recurring feature of the pre-MEK era is that agents which delayed progression did not shrink tumors: peginterferon alfa-2b more than doubled the time to progression relative to the tipifarnib placebo arm (29.4 versus 11.8 months) yet produced a ≥ 20% volume reduction in only 4 of 83 patients, and clinical improvement bore little relation to imaging change [13]. Sirolimus added 3.5 months to the time to progression without producing responses [12]. Tipifarnib [10] and pirfenidone [11] did neither, their largest observed volume decreases being 11.3% and 12%. Imatinib shrank tumors in 14% of patients, almost entirely lesions under 20 mL [14].

**Table 3:** Non-MEK targeted agents evaluated in NF1-associated plexiform neurofibroma, in approximate chronological order.

Agent	Target/class	Design	N	Outcome
Tipifarnib [10]	Farnesyltransferase inhibitor	Randomized, placebo-controlled phase 2	60	No prolongation of time to progression versus placebo. Maximum volume decrease observed in any patient was 11.3%. Its placebo arm (median TTP 10.6–11.8 months) became the historical control for later single-arm trials
Pirfenidone [11]	Antifibrotic	Phase 2	36	No effect on time to progression; largest volume decrease 12%
Sirolimus [12]	mTOR inhibitor	Phase 2 (two strata)	—	Time to progression prolonged by ~3.5 months versus the tipifarnib placebo arm; objective responses were not seen
Peginterferon alfa-2b [13]	Immunomodulatory/antiangiogenic	Phase 2 (three strata)	84	Median TTP 29.4 months in the progressive stratum versus 11.8 months for placebo (p = 0.031) — more than double. Volumetric responses were rare (4/83, 5%), and clinical improvement correlated poorly with imaging
Imatinib [14]	KIT/PDGFR inhibitor	Phase 2	36	≥20% volume reduction in 5/36 (14%), essentially confined to tumors <20 mL
Cabozantinib [26]	Multi-receptor tyrosine kinase inhibitor (MET, VEGFR2, AXL)	Phase 2, Simon two-stage (NCT02101736)	19^a^	PR in 8/19 (42%); median volume change −15.2%; no patient progressed on treatment. Responders had significant reductions in pain intensity and interference. Grade 3 events in 8 patients; 9 required dose reduction or stopped. The first non-MEK agent with response rates approaching MEK inhibition
HLX-1502 [49]	Oral small molecule, non-MEK mechanism	Phase 2, open-label, single-arm (INSPIRE-NF1/NF119; NCT06541847)	20 (planned)	Currently enrolling patients ≥16 years with progressive or symptomatic PN; first patient dosed February 2025. Endpoints are tumor response rate, safety and pharmacokinetics. Positioned as a potential alternative to MEK inhibition with a different tolerability profile

^a^Efficacy-evaluable population.

PN, plexiform neurofibroma; PR, partial response; TTP, time to progression.

Cabozantinib is the notable exception. In a Simon two-stage phase 2 trial in patients aged 16 and above, 8 of 19 evaluable participants (42%) achieved a partial response, no patient progressed on treatment, and responders reported significant reductions in pain intensity and interference, a response rate indistinguishable from that of mirdametinib in the same age band [18,26,41], achieved by inhibiting the tumor microenvironment rather than MAPK signaling in the Schwann cell lineage. Its toxicity differs accordingly, with hypothyroidism and palmar-plantar erythrodysesthesia rather than rash and paronychia, and it was less well tolerated, with 9 of 19 patients requiring dose reduction or discontinuation [26]. Having two mechanistically distinct classes with comparable activity opens the way to rational sequencing and to combination; a phase 1/1b/2 study of cabozantinib with selumetinib in adults and adolescents (NF113, NCT06502171) is now open [50].

The pipeline now extends beyond MEK. HLX-1502, an orally administered small molecule discovered through a machine-learning platform and acting outside the MEK class, entered a phase 2 open-label single-arm study (INSPIRE-NF1/NF119, NCT06541847). It is enrolling approximately 20 participants aged 16 and above with progressive or symptomatic plexiform neurofibroma, with tumor response rate, safety and pharmacokinetics as endpoints [49].

### Strengths and limitations

This review searched four databases and two registries, applied duplicate independent screening and extraction, appraised bias with design-appropriate instruments, and quantified response with a pre-specified random-effects model and a subgroup hypothesis that proved explanatory. To our knowledge it is the first synthesis to pool tumor response across all four MEK inhibitors studied in this disease, to separate pediatric from adult effects formally, and to incorporate both the randomized adult evidence and the mirdametinib registration data.

Its limitations are substantive. The protocol was not prospectively registered, which weakens the safeguard against selective outcome reporting; we have mitigated this by reporting every pre-specified analysis, including the sensitivity analysis that attenuated our headline estimate. Eight of the nine pooled strata are single-arm and open-label, so the pooled proportion cannot be read as a treatment effect: spontaneous arrest of tumor growth occurs in this disease [6], and only KOMET quantifies the counterfactual directly, at 5% [21]; the SPRINT propensity-score analysis provides an indirect external comparison for pediatric selumetinib only [44] and does not extend to mirdametinib, trametinib or binimetinib in either age group. The low statistical heterogeneity of the pediatric estimate (I^2^ = 17.4%) should therefore be read as consistency across uncontrolled cohorts, not as evidence of a treatment effect: the majority of the 62.9% pediatric figure has no concurrent comparator arm. Two included reports are congress abstracts pending full publication [24,40]. Two of the four agents contribute a single study each, so agent-level estimates are fragile. Publication bias could not be formally tested with nine strata, and small early-phase studies with negative findings are unlikely to have reached publication. Finally, follow-up for mirdametinib and for the adult indications remains short relative to the duration of therapy these patients face.

### Implications for practice and research

For practice, these data support offering MEK inhibition to children and adults with symptomatic, inoperable plexiform neurofibroma, with the expectation of partial shrinkage in roughly six of ten children and four of ten adults, and of symptomatic benefit in a broader group. The six-in-ten pediatric estimate, however, is derived predominantly from uncontrolled single-arm cohorts rather than from controlled comparisons, and this uncertainty should be conveyed when counselling families. Patients should be counselled that a response means a reduction of roughly a quarter of tumor volume rather than resolution, and that benefit depends on continued treatment. Baseline echocardiography, ophthalmologic examination, and creatine phosphokinase and hepatic panels should be obtained and repeated at intervals for as long as therapy continues. Radiographic surveillance should remain alert to distinct nodular lesions, which may signal atypical transformation and warrant separate evaluation rather than being read as part of the treated plexiform mass [48]. Response should be assessed volumetrically, as RECIST underestimates response in this disease by more than half and should not be used [24]. Pain and function deserve consideration as outcomes, and an agent that controls symptoms should not be withdrawn solely because a volumetric threshold has not been crossed.

For research, the priorities are a randomized trial of continued versus interrupted therapy in responders, harmonized patient-reported endpoints, comparative or platform designs across MEK inhibitors and against cabozantinib, long-term registries linked to cancer data that capture atypical transformation and not only frank malignancy, and the identification of predictive biomarkers, which remain absent. Neither NF1 genotype nor baseline tumor volume reliably distinguishes responders, and pharmacokinetic exposure explained only a fraction of the response variance in NF106 [41].

## Conclusions

A disease that had no effective systemic therapy in 2015 now has two approved drugs spanning infancy to adulthood, further agents with demonstrated activity, randomized evidence in adults, and a mechanistically distinct competitor in cabozantinib. Pooled across nine study strata, the descriptive volumetric response rate was 62.9% in children and 40.8% in adults; these are weighted averages across four pharmacologically distinct agents, and only the adult estimate is anchored by a randomized comparator. Benefit is durable and the toxicity profile predictable and manageable, though responses are partial and treatment is suppressive rather than curative. The questions that remain are how long to treat, what to do when treatment stops, whether these drugs prevent malignant transformation, and how to measure benefit in a way that reflects what patients experience. With current studies and data collection ongoing, further insight should help to answer them.

## Supporting information

S1 File Full electronic search strategies for MEDLINE (PubMed), Embase, CENTRAL, Web of Science, ClinicalTrials.gov and the WHO ICTRP, with the number of records retrieved from each source. (DOCX)

S2 File Completed PRISMA 2020 checklist. (DOCX)

S3 Table Report and data cut selected for each unique study and pooled stratum, showing enrolled and evaluable numbers and how overlapping cohorts were reconciled to avoid double-counting. (DOCX)

S4 Table Post hoc subgroup analysis of objective response by agent within the adult stratum, with within- and between-agent heterogeneity. (DOCX)
