# Supplementary material for: Efficacy and safety of MEK inhibitors for NF1-associated symptomatic, inoperable plexiform neurofibromas: A systematic review and meta-analysis
Source: PLoS One. 2026 Oct 6;21(10):e0359957. doi: 10.1371/journal.pone.0359957 (PMC13641391; doi:10.1371/journal.pone.0359957)
Supplement: Supplementary file 1 [file pone.0359957.s001.docx]

# Full Electronic Search Strategies

All searches were run from database inception to **10 July 2026**. Controlled vocabulary (MeSH, Emtree) was combined with free-text terms across three concept blocks: (i) neurofibromatosis type 1, (ii) plexiform neurofibroma, and (iii) MEK1/2 inhibitors, including the class term and the four named agents together with their development codes. No language, date or publication-type limits were applied. Trial registries and conference proceedings were searched separately (Sects. 6 and 7). Results were exported to EndNote and de-duplicated before screening.

**Concept structure common to all databases:**

Block A (population 1): neurofibromatosis type 1, NF1, von Recklinghausen disease.

Block B (population 2): plexiform neurofibroma(s), plexiform neurofibromata.

Block C (intervention): MEK inhibitor(s), MAP2K1/MAP2K2 inhibitor; selumetinib (AZD6244, ARRY-142886); mirdametinib (PD-0325901); trametinib (GSK1120212, JTP-74057); binimetinib (MEK162, ARRY-438162, ARRY-162).

*Final line: (A OR B) AND C.*

## 1 Records retrieved by source

| **Source** | **Records** |
| --- | --- |
| MEDLINE (via PubMed) | 312 |
| Embase (via Elsevier) | 468 |
| Cochrane CENTRAL | 89 |
| Web of Science Core Collection | 198 |
| ***Subtotal — bibliographic databases*** | **1,067** |
| ClinicalTrials.gov | 41 |
| WHO ICTRP | 25 |
| ***Subtotal — trial registries*** | **66** |
| ***Total before de-duplication*** | **1,133** |

*After removal of 389 duplicates, 744 unique records proceeded to title/abstract screening (Fig. 1 of the main text).*

## 2 MEDLINE (PubMed)

*Interface: PubMed. Field tags: [mh] = MeSH heading, [tiab] = title/abstract, [nm] = substance name. Date run: 10 July 2026.*

**#1** "Neurofibromatosis 1"[mh]

**#2** ("neurofibromatosis type 1"[tiab] OR "neurofibromatosis 1"[tiab] OR NF1[tiab] OR "von Recklinghausen"[tiab])

**#3** #1 OR #2

**#4** "Neurofibroma, Plexiform"[mh]

**#5** ("plexiform neurofibroma"[tiab] OR "plexiform neurofibromas"[tiab] OR "plexiform neurofibromata"[tiab])

**#6** #4 OR #5

**#7** #3 OR #6

**#8** "MAP Kinase Kinase 1"[mh] OR "MAP Kinase Kinase 2"[mh]

**#9** ("MEK inhibitor"[tiab] OR "MEK inhibitors"[tiab] OR "MEK1/2 inhibitor"[tiab] OR "MAP2K inhibitor"[tiab])

**#10** (selumetinib[tiab] OR selumetinib[nm] OR AZD6244[tiab] OR "ARRY-142886"[tiab] OR Koselugo[tiab])

**#11** (mirdametinib[tiab] OR "PD-0325901"[tiab] OR PD0325901[tiab] OR Gomekli[tiab])

**#12** (trametinib[tiab] OR trametinib[nm] OR GSK1120212[tiab] OR "JTP-74057"[tiab] OR Mekinist[tiab])

**#13** (binimetinib[tiab] OR binimetinib[nm] OR MEK162[tiab] OR "ARRY-438162"[tiab] OR "ARRY-162"[tiab] OR Mektovi[tiab])

**#14** #8 OR #9 OR #10 OR #11 OR #12 OR #13

**#15** #7 AND #14

**Records retrieved: 312.**

## 3 Embase (Elsevier)

*Interface: Embase.com. Field suffixes: /exp = Emtree term exploded, :ti,ab,kw = title/abstract/keyword. Date run: 10 July 2026.*

**#1** 'neurofibromatosis type 1'/exp

**#2** ('neurofibromatosis type 1':ti,ab,kw OR 'neurofibromatosis 1':ti,ab,kw OR nf1:ti,ab,kw OR 'von recklinghausen':ti,ab,kw)

**#3** #1 OR #2

**#4** 'plexiform neurofibroma'/exp

**#5** ('plexiform neurofibroma':ti,ab,kw OR 'plexiform neurofibromas':ti,ab,kw OR 'plexiform neurofibromata':ti,ab,kw)

**#6** #4 OR #5

**#7** #3 OR #6

**#8** 'mitogen activated protein kinase kinase inhibitor'/exp

**#9** ('mek inhibitor':ti,ab,kw OR 'mek1/2 inhibitor':ti,ab,kw OR 'map2k inhibitor':ti,ab,kw)

**#10** 'selumetinib'/exp OR selumetinib:ti,ab,kw OR azd6244:ti,ab,kw OR 'arry 142886':ti,ab,kw OR koselugo:ti,ab,kw

**#11** 'mirdametinib'/exp OR mirdametinib:ti,ab,kw OR 'pd 0325901':ti,ab,kw OR pd0325901:ti,ab,kw OR gomekli:ti,ab,kw

**#12** 'trametinib'/exp OR trametinib:ti,ab,kw OR gsk1120212:ti,ab,kw OR 'jtp 74057':ti,ab,kw OR mekinist:ti,ab,kw

**#13** 'binimetinib'/exp OR binimetinib:ti,ab,kw OR mek162:ti,ab,kw OR 'arry 438162':ti,ab,kw OR 'arry 162':ti,ab,kw OR mektovi:ti,ab,kw

**#14** #8 OR #9 OR #10 OR #11 OR #12 OR #13

**#15** #7 AND #14

**Records retrieved: 468.**

## 4 Cochrane Central Register of Controlled Trials (CENTRAL)

*Interface: Cochrane Library (Wiley). MeSH descriptors exploded; free text in Title/Abstract/Keyword. Date run: 10 July 2026.*

**#1** MeSH descriptor: [Neurofibromatosis 1] explode all trees

**#2** ("neurofibromatosis type 1" OR "neurofibromatosis 1" OR NF1):ti,ab,kw

**#3** MeSH descriptor: [Neurofibroma, Plexiform] explode all trees

**#4** ("plexiform neurofibroma*"):ti,ab,kw

**#5** #1 OR #2 OR #3 OR #4

**#6** (selumetinib OR AZD6244 OR mirdametinib OR "PD-0325901" OR trametinib OR GSK1120212 OR binimetinib OR MEK162 OR "MEK inhibitor*"):ti,ab,kw

**#7** #5 AND #6

**Records retrieved: 89.**

## 5 Web of Science Core Collection

*Interface: Clarivate. Field tag: TS = Topic (title, abstract, author keywords, Keywords Plus). Date run: 10 July 2026.*

TS = (

("neurofibromatosis type 1" OR "neurofibromatosis 1" OR NF1 OR

"von Recklinghausen" OR "plexiform neurofibroma*")

) AND TS = (

("MEK inhibitor*" OR "MEK1/2 inhibitor*" OR selumetinib OR AZD6244 OR

"ARRY-142886" OR mirdametinib OR "PD-0325901" OR trametinib OR

GSK1120212 OR binimetinib OR MEK162 OR "ARRY-438162")

)

**Records retrieved: 198.**

## 6 Trial registries

**ClinicalTrials.gov** — searched via the Advanced Search interface using the condition and intervention fields:

Condition/disease: "plexiform neurofibroma" OR "neurofibromatosis type 1"

Intervention/treatment: selumetinib OR mirdametinib OR trametinib OR

binimetinib OR "MEK inhibitor"

**Records retrieved: 41.**

**WHO ICTRP** — searched in the standard search using the same condition/intervention combinations; the ICTRP aggregates non-US primary registries (EU-CTR, ISRCTN, ANZCTR, JPRN and others).

Condition: plexiform neurofibroma OR neurofibromatosis type 1

Intervention: selumetinib OR mirdametinib OR trametinib OR binimetinib OR

MEK inhibitor

**Records retrieved: 25. Registry entries were cross-checked against published reports to avoid double-counting completed trials.**

## 7 Conference proceedings and other sources

The following were hand-searched for the five years preceding the search date (2021–2026):

- American Society of Clinical Oncology (ASCO) Annual Meeting abstracts
- Society for Neuro-Oncology (SNO) Annual Meeting abstracts
- Children's Tumor Foundation (CTF) NF Conference abstracts

In addition, the reference lists of all included studies and of prior systematic reviews were screened, and study investigators were contacted where reported outcome data appeared incomplete. Congress abstracts were eligible only where they provided extractable outcome data not yet available in a full-text publication.
