# Supplementary material for: Efficacy and safety of MEK inhibitors for NF1-associated symptomatic, inoperable plexiform neurofibromas: A systematic review and meta-analysis
Source: PLoS One. 2026 Oct 6;21(10):e0359957. doi: 10.1371/journal.pone.0359957 (PMC13641391; doi:10.1371/journal.pone.0359957)
Supplement: Supplementary file 2 [file pone.0359957.s002.docx]

**PRISMA 2020 Checklist**

Reporting follows the PRISMA 2020 statement (Page MJ, et al. *BMJ* 2021;372:n71). "Location" gives the section, table or figure of the main manuscript, or the Online Resource, where each item is reported. Section numbers refer to the numbered headings of the main text.

| **Section / Topic** | **Item** | **Checklist item** | **Location** |
| --- | --- | --- | --- |
| **TITLE** | | | |
| Title | **1** | Identify the report as a systematic review. | Title page (p. 1) |
| **ABSTRACT** | | | |
| Abstract | **2** | See the PRISMA 2020 for Abstracts checklist. | Abstract, structured (p. 2) |
| **INTRODUCTION** | | | |
| Rationale | **3** | Describe the rationale for the review in the context of existing knowledge. | Sect. 1 (p. 3) |
| Objectives | **4** | Provide an explicit statement of the objective(s) or question(s) the review addresses. | Sect. 1, final para. (p. 4); Abstract (p. 2) |
| **METHODS** | | | |
| Eligibility criteria | **5** | Specify the inclusion and exclusion criteria and how studies were grouped for the syntheses. | Sect. 2.2 (p. 5) |
| Information sources | **6** | Specify all databases, registers and other sources searched, with the date each was last searched. | Sect. 2.3 (p. 6); Online Resource 1 |
| Search strategy | **7** | Present the full search strategies for all databases, registers and websites, including filters and limits. | Online Resource 1 |
| Selection process | **8** | Specify the methods used to decide whether a study met the inclusion criteria, including reviewers and any automation. | Sect. 2.4 (p. 6) |
| Data collection process | **9** | Specify the methods used to collect data from reports, including number of reviewers and any automation. | Sect. 2.4 (p. 6) |
| Data items | **10a** | List and define all outcomes for which data were sought; state whether all results were sought. | Sect. 2.2 (p. 5), 2.4 (p. 6) |
| Data items | **10b** | List and define all other variables for which data were sought (e.g., participant and intervention characteristics). | Sect. 2.4 (p. 6); Table 1 (p. 22) |
| Study risk of bias assessment | **11** | Specify the methods used to assess risk of bias, including the tool(s) and number of reviewers. | Sect. 2.4 (p. 6) |
| Effect measures | **12** | Specify the effect measure(s) used in the synthesis or presentation of results. | Sect. 2.5 (p. 7) |
| Synthesis methods | **13a** | Describe the processes used to decide which studies were eligible for each synthesis. | Sect. 2.5 (p. 7) |
| Synthesis methods | **13b** | Describe methods to prepare the data for presentation or synthesis (e.g., handling of missing statistics, conversions). | Sect. 2.5 (p. 7) |
| Synthesis methods | **13c** | Describe methods used to tabulate or visually display results of individual studies and syntheses. | Sect. 2.5 (p. 7); Table 1 (p. 22); Fig 2 (p. 36) |
| Synthesis methods | **13d** | Describe methods to synthesise results and rationale; if meta-analysis, identify model, packages and statistics. | Sect. 2.5 (p. 7) |
| Synthesis methods | **13e** | Describe methods used to explore possible causes of heterogeneity (e.g., subgroup analysis, meta-regression). | Sect. 2.5, age subgroup (p. 7) |
| Synthesis methods | **13f** | Describe any sensitivity analyses conducted to assess robustness of synthesised results. | Sect. 2.5, RCT removed (p. 7) |
| Reporting bias assessment | **14** | Describe methods used to assess risk of bias due to missing results in a synthesis. | Sect. 2.5, not formally assessed, <10 studies (p. 7) |
| Certainty assessment | **15** | Describe methods used to assess certainty (or confidence) in the body of evidence for an outcome. | Sect. 2.4, GRADE (p. 6) |
| **RESULTS** | | | |
| Study selection | **16a** | Describe results of the search and selection, from records identified to studies included, ideally using a flow diagram. | Sect. 3.1 (p. 8); Fig 1 (p. 36) |
| Study selection | **16b** | Cite studies that met many but not all inclusion criteria and explain why they were excluded. | Sect. 3.1 (p. 8) |
| Study characteristics | **17** | Cite each included study and present its characteristics. | Sect. 3.2 (p. 8); Table 1 (p. 22) |
| Risk of bias in studies | **18** | Present assessments of risk of bias for each included study. | Sect. 3.7 (p. 11) |
| Results of individual studies | **19** | For all outcomes, present for each study summary statistics and an effect estimate with precision, ideally structured. | Table 1 (p. 22); Fig 2 (p. 36) |
| Results of syntheses | **20a** | For each synthesis, briefly summarise the characteristics and risk of bias among contributing studies. | Sect. 3.2, 3.3 (p. 8), 3.7 (p. 11) |
| Results of syntheses | **20b** | Present results of all statistical syntheses; if meta-analysis, present summary estimate, precision and heterogeneity. | Sect. 3.3 (p. 8); Fig 2 (p. 36) |
| Results of syntheses | **20c** | Present results of all investigations of possible causes of heterogeneity among study results. | Sect. 3.3, age subgroups (p. 8) |
| Results of syntheses | **20d** | Present results of all sensitivity analyses conducted to assess robustness of synthesised results. | Sect. 3.3, KOMET excluded (p. 9) |
| Reporting biases | **21** | Present assessments of risk of bias due to missing results for each synthesis assessed. | Sect. 2.5 (p. 7), 3.7 (p. 11), not assessed |
| Certainty of evidence | **22** | Present assessments of certainty (or confidence) in the body of evidence for each outcome assessed. | Sect. 3.7, GRADE (p. 11) |
| **DISCUSSION** | | | |
| Discussion | **23a** | Provide a general interpretation of the results in the context of other evidence. | Sect. 4.1–4.7 (pp. 13–17) |
| Discussion | **23b** | Discuss any limitations of the evidence included in the review. | Sect. 4.8 (p. 18) |
| Discussion | **23c** | Discuss any limitations of the review processes used. | Sect. 4.8 (p. 18) |
| Discussion | **23d** | Discuss implications of the results for practice, policy and future research. | Sect. 4.9 (p. 19) |
| **OTHER INFORMATION** | | | |
| Registration and protocol | **24a** | Provide registration information, including the register name and registration number, or state not registered. | Sect. 2.1, not registered with PROSPERO (p. 4) |
| Registration and protocol | **24b** | Indicate where the review protocol can be accessed, or state that a protocol was not prepared. | Sect. 2.1 (p. 4); available from author |
| Registration and protocol | **24c** | Describe and explain any amendments to information provided at registration or in the protocol. | Not applicable (no amendments) |
| Support | **25** | Describe sources of financial or non-financial support and the role of funders. | Declarations, Funding (p. 27) |
| Competing interests | **26** | Declare any competing interests of review authors. | Declarations, Competing interests (p. 27) |
| Availability of data, code and materials | **27** | Report which of the following are publicly available and where: data, analytic code, other materials. | Declarations, Data availability (p. 27); Online Resources 1, 2 |

*From: Page MJ, McKenzie JE, Bossuyt PM, et al. The PRISMA 2020 statement: an updated guideline for reporting systematic reviews. BMJ 2021;372:n71. doi:10.1136/bmj.n71. For more information, visit http://www.prisma-statement.org/.*
