# Supplementary material for: Efficacy and safety of MEK inhibitors for NF1-associated symptomatic, inoperable plexiform neurofibromas: A systematic review and meta-analysis
Source: PLoS One. 2026 Oct 6;21(10):e0359957. doi: 10.1371/journal.pone.0359957 (PMC13641391; doi:10.1371/journal.pone.0359957)
Supplement: Supplementary file 3 [file pone.0359957.s003.docx]

**S3 Table. Report and data cut selected for each unique study and pooled stratum.**

For each of the nine unique studies, the report and data cut used for the meta-analysis of objective response are shown, together with enrolled and evaluable numbers and the rules applied to reconcile overlapping cohorts. Where a study generated more than one report (SPRINT; ReNeu long-term follow-up), the study was counted once toward the unique-study and enrolled-participant totals, and only the report indicated here contributed to pooling. Where a single trial reported separate pediatric and adult strata (ReNeu), each stratum contributed to pooling once while the trial was counted once toward unique-study totals.

| **Unique study (trial)** | **Agent** | **Report / data cut selected for pooling** | **Enrolled N** | **Evaluable N (pooled)** | **Accounting notes** |
| --- | --- | --- | --- | --- | --- |
| SPRINT (NCT01362803) | Selumetinib | Gross 2020 (SPRINT phase 2), pediatric ORR by independent central review [15] | 50 | 50 | Reported across three linked reports: Dombi 2016 phase 1 (N=24) [14], Gross 2020 phase 2 (N=50) [15], and Gross 2023 long-term follow-up (N=74) [19]. Counted once. The phase 2 report [15] supplied the pooled pediatric response stratum; the long-term follow-up N of 74 was used for the enrolled-participant total to capture the fullest cohort. Dombi 2016 and Gross 2023 retained in narrative synthesis only. |
| PNOC010 / NF108 (NCT03231306) | Binimetinib | Mueller 2020 (conference abstract) [30] | 19 | 19 | Efficacy-evaluable population; enrolment larger. Abstract-only data — no full peer-reviewed publication available. |
| TRAM-01 (NCT03363217) | Trametinib | Perreault 2022 (conference abstract) [29] | 28 | 28 | Efficacy-evaluable population. Volumetric ORR used for pooling; RECIST 1.1 result not pooled. |
| McCowage 2018 (NCT02124772) | Trametinib | McCowage 2018 [28] | 26 | 26 | Response by independent volumetric review used for pooling. |
| ReNeu (NCT03962543) — pediatric | Mirdametinib | Moertel 2025, pediatric stratum, BICR-confirmed [24] | 56 | 56 | ReNeu enrolled both children and adults and reported them separately; each age stratum entered pooling once. Primary publication [24] used; long-term follow-up [26] retained in narrative only. |
| NF106 (NCT02096471) | Mirdametinib | Weiss 2021 [27] | 19 | 19 | Adult/adolescent stratum. PR by course 12 used for pooling. |
| Adult selumetinib phase 2 | Selumetinib | Gross 2025 [21] | 33 | 33 | Adult single-arm stratum. |
| ReNeu (NCT03962543) — adult | Mirdametinib | Moertel 2025, adult stratum, BICR-confirmed [24] | 58 | 58 | Adult stratum of ReNeu, counted separately from the pediatric stratum above (both derive from the same 114-patient trial; the trial is counted once toward unique-study totals). |
| KOMET (NCT04924608) | Selumetinib | Chen 2025 [22] | 145 | 71 | Randomized, placebo-controlled. Only the selumetinib arm (N=71 evaluable) contributed to the single-arm-comparable ORR pool; the 145 enrolled spans both arms. |
| Italian multicenter cohort | Selumetinib | Italian retrospective cohort 2025 [34] | 70 | — | Retrospective real-world cohort; retained in narrative synthesis, excluded from pooling to avoid mixing prospective and retrospective designs. |

*BICR, blinded independent central review; ORR, objective response rate; PR, partial response; RECIST, Response Evaluation Criteria in Solid Tumors.*
