# Supplementary material for: Efficacy and safety of MEK inhibitors for NF1-associated symptomatic, inoperable plexiform neurofibromas: A systematic review and meta-analysis
Source: PLoS One. 2026 Oct 6;21(10):e0359957. doi: 10.1371/journal.pone.0359957 (PMC13641391; doi:10.1371/journal.pone.0359957)
Supplement: Supplementary file 4 [file pone.0359957.s004.docx]

**S4 Table. Post hoc subgroup analysis of objective response by agent within the adult stratum.**

The adult stratum is the only one in which two agents are each represented by more than one study, making an agent subgroup test interpretable. Proportions were pooled with the same Freeman–Tukey random-effects model used throughout. Between-agent heterogeneity was tested with Cochran’s Q.

**All adult studies**

| **Agent (adult stratum)** | **Studies (n)** | **ORR** | **Within-agent I²** | **Contributing studies** |
| --- | --- | --- | --- | --- |
| Selumetinib | 2 | 40.1% | 94.7% | Gross 2025 (63.6%); KOMET (19.7%) |
| Mirdametinib | 2 | 41.5% | 0.0% | NF106 (42.1%); ReNeu adult (41.4%) |

*Between-agent difference: Q = 0.00, df = 1, p = 0.95 (not significant). The within-selumetinib I² of 94.7% reflects the design contrast between KOMET (fixed cycle-16 assessment, blinded central review) and the single-arm adult study, not agent identity.*

**Excluding KOMET**

| **Agent** | **Studies (n)** | **ORR** | **Note** |
| --- | --- | --- | --- |
| Selumetinib | 1 | 63.6% | Gross 2025 only (KOMET removed) |
| Mirdametinib | 2 | 41.5% | Unchanged |

*Between-agent difference: Q = 4.40, df = 1, p = 0.036. This apparent divergence rests on a single selumetinib study and is confounded by assessment window; it should not be read as a true agent effect. A meta-regression on agent across all nine strata was not performed, because two of the four agents contribute a single stratum each and agent is partly confounded with age, leaving the model over-parameterized.*

*ORR, objective response rate. Values computed under the manuscript’s Freeman–Tukey double-arcsine random-effects model (variance 1/(4n+2)), which reproduces the published adult estimates of 40.8% (I² = 85.3%) and, excluding KOMET, 49.0% (I² = 54.6%).*
